# Philadelphia Dental College

**Published:** 1869-04

**Authors:** 


### ARTICLE VIII.

*Philadelphia Dental College.*

The Sixth Annual Commencement of this College was held at Horticultural Hall, February 26th.

The Valedictory Address was delivered by Professor Harrison Allen. The degree of "Doctor of Dental Surgery" was conferred upon the following gentlemen, twenty-five in number:

Paul Emilio Besse, *Cuba* ; James A. Blum, *North Carolina ;* Alonzo Boice, *New York ;* H. Edmond Casgrain, *Canada ;* James M. Caufman, *Pennsylvania ;* Alfred C. Cogswell, *Nova Scotia ;* Varnum D. Collins, *China ;* Brooke Davis, *Pennsylvania ;* Henry N. Dodge, M. D., *New York* ; Luis Estrada, *Cuba ;* Joseph Holmes, *Ohio ;* Henry M. Humphrey, *Massachusetts ;* Absalom M. Jarrett, *West Virginia ;* Henry W. Ladd, *Maine ;* Oscar P. Macalaster, *Nova Scotia ;* Isaac N. McCuddy, *Kentucky ;* Newton Morgan, *Massachusetts ;* Judson N. Niles, *Vermont ;* Benjamin Percival, Jr., *Massachusetts ;* Adolf Petermann, *Germany ;* Charles E. Pike, *Maine ;* Albert J. Snead, *Virginia ;* Mordaunt Stevens, *France ;* Thomas G. Wardle, *Pennsylvania ;* Henry M. Welch, *New York.*

